# Acute Myeloid Leukemia Stem Cells: The Challenges of Phenotypic Heterogeneity

**DOI:** 10.3390/cancers12123742

**Published:** 2020-12-12

**Authors:** Marlon Arnone, Martina Konantz, Pauline Hanns, Anna M. Paczulla Stanger, Sarah Bertels, Parimala Sonika Godavarthy, Maximilian Christopeit, Claudia Lengerke

**Affiliations:** 1 Department of Biomedicine, University of Basel and University Hospital Basel, Hebelstrasse 20, 4031 Basel, Switzerland; marlon.arnone@unibas.ch (M.A.); martina.konantz@unibas.ch (M.K.); pauline.hanns@unibas.ch (P.H.); 2 Internal Medicine II, Hematology, Oncology, Clinical Immunology and Rheumatology, Department for Internal Medicine, University Hospital Tübingen, Otfried-Müller-Str. 10, 72076 Tübingen, Germany; anna.stanger@med.uni-tuebingen.de (A.M.P.S.); sarah.bertels@med.uni-tuebingen.de (S.B.); Parimala.Godavarthy@med.uni-tuebingen.de (P.S.G.); Maximilian.Christopeit@med.uni-tuebingen.de (M.C.)

**Keywords:** acute myeloid leukemia, leukemic stem cells, cellular heterogeneity, markers, relapse

## Abstract

**Simple Summary:**

Relapse after apparent remission remains a major cause of death in patients with acute myeloid leukemia (AML). On the cellular level, leukemia relapse is considered to emerge from subpopulations of therapy-resistant leukemic stem cells (LSC). Identification and targeting of LSC are thus most important goals for AML treatment. However, AML and their LSC are highly heterogeneous. Here, we review the current knowledge on AML LSC identification and targeting via surface antigens with a focus on heterogeneity among different AML subgroups and genetic backgrounds.

**Abstract:**

Patients suffering from acute myeloid leukemia (AML) show highly heterogeneous clinical outcomes. Next to variabilities in patient-specific parameters influencing treatment decisions and outcome, this is due to differences in AML biology. In fact, different genetic drivers may transform variable cells of origin and co-exist with additional genetic lesions (e.g., as observed in clonal hematopoiesis) in a variety of leukemic (sub)clones. Moreover, AML cells are hierarchically organized and contain subpopulations of more immature cells called leukemic stem cells (LSC), which on the cellular level constitute the driver of the disease and may evolve during therapy. This genetic and hierarchical complexity results in a pronounced phenotypic variability, which is observed among AML cells of different patients as well as among the leukemic blasts of individual patients, at diagnosis and during the course of the disease. Here, we review the current knowledge on the heterogeneous landscape of AML surface markers with particular focus on those identifying LSC, and discuss why identification and targeting of this important cellular subpopulation in AML remains challenging.

## 1. Introduction

Acute myeloid leukemia (AML) is a devastating, rapidly-evolving disease characterized by an abnormal proliferation of poorly-differentiated cells which impairs normal hematopoiesis. AML patients suffer from cytopenia associated with recurrent infections, anemia, easy bleeding, and other manifestations [1] and show highly variable responses to therapy and survival rates. Notably, a major cause of disease progression and relapse is the persistence of therapy-resistant, clonogenic leukemic subpopulations: the leukemic stem cells (LSC) [2].

In 1994, John Dick and colleagues were the first to prove the existence of human LSC in an in vivo experimental model. Human CD34^+^ leukemic cells were shown to repopulate the bone marrow (BM) of severe combined immunodeficient (SCID) mice, while CD34^−^ leukemic blasts remained non-leukemogenic [3,4]. These CD34^+^ cells responsible for leukemia initiation and maintenance were termed LSC. Nowadays, they are documented as cells with enhanced capacities to selectively escape chemotherapy treatments [5] as well as immune surveillance [6], thus leading to disease relapse after therapy, a major cause of death in these patients. Since AML is highly heterogeneous with respect to genetic alterations, epigenetics, and leukemia cell of origin, it is not surprising that considerable heterogeneity is also observed among surface markers of AML cells and their LSC [2], making immunological targeting of LSC a constant challenge [7,8,9].

## 2. Leukemic Stem Cells and Healthy Stem/Progenitor Cells 

Hematopoiesis is organized hierarchically with a minor subset of hematopoietic stem cells (HSC) giving rise to all blood cells during the lifespan of an individual. HSC must balance regenerative requirements (which naturally involve cell division and differentiation) with the need to protect their own genomic integrity by reducing cell division. In order to achieve this, HSC undergo highly complex fine-tuned interactions with the BM microenvironment and interact with several other cell types (e.g., osteoblasts, stromal cells, endothelial cells, adipocytes, and neural cells) via soluble factors, biophysical forces, and cell-mediated interactions [10]. Similarly, LSC also reside and are influenced by the so-called BM niche, which sustains their quiescence and protects them from genotoxic stress [11,12].

AML is also organized hierarchically and contains subpopulations of LSC that share functional and molecular properties with their cells of origin, the healthy hematopoietic stem and progenitor cells (HSPC) [4,13,14,15]. Consistent with a close relationship between these two cell types, molecules expressed on healthy HSPC, i.e., CD34, were also reported to identify LSC [16]. Functionally, the CD34 family encompasses podocalyxin and endoglycan proteins and is considered to regulate cell differentiation, adhesion, trafficking, and proliferation [17]. CD34 is expressed on the vast majority of HSC, but rare CD34^−^ HSC giving rise to CD34^+^ HSPC have also been reported [16]. 

In 2016, the LSC17 gene expression score was defined as the molecular LSC hallmark that predicts outcome and treatment resistance in patients with AML [18]. Among the genes highlighted in the LSC17 score were e.g., CD34 and the G protein-coupled receptor GPR56, a surface protein involved in cell adhesion which was also described to mark healthy HSC [16,19]. However, great phenotypic heterogeneity is observed in AML LSC and a wide range of surface markers has been found to identify LSC in only some, but not all AML (e.g., CD93, TIM3, CD44, CD123, etc. [9,20,21,22,23,24,25,26]).

## 3. The Relevance of Immunomodulatory Proteins for LSC Detection

**Figure 1:**
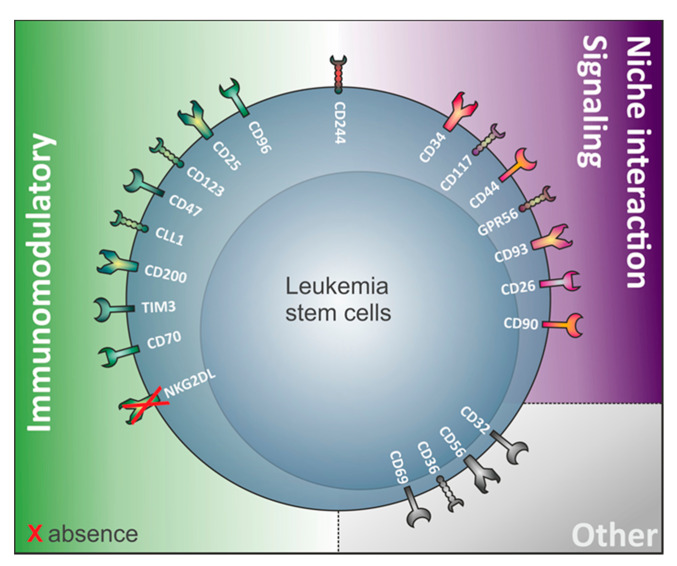
Surface antigens involved in leukemic stem cells (LSC) identification. Several surface proteins involved in LSC identification are involved in immune processes (e.g., TIM-3, CLL-1, CD47…) or interactions with the bone marrow niche (e.g., GPR56, CD44…).

Interestingly, a variety of antigens involved in LSC identification are in fact involved in immunological processes (Figure 1, Table 1). This suggests that LSC and non-LSC may have different interactions with the immune system. This notion has been substantiated by recent work from our research group demonstrating that LSC selectively escape immune surveillance by suppressing surface expression of NKG2D ligands (NKG2DL) [6]. When compared to corresponding non-stem leukemic blasts from the same patients, LSC lack expression of such ligands for activating NKG2D receptors on natural killer (NK) cells thereby evading NK-mediated lysis. In several AML patient samples of heterogeneous genetic backgrounds, lack of NKG2DL surface expression robustly distinguished LSC from other non-stem leukemic cells [6].

Other examples of immunomodulatory proteins involved in LSC identification (Figure 1) include the immunoglobulin superfamily member CD96, a molecule expressed on healthy T and natural killer cells with known inhibitory roles on NK cells [27], TIM-3 (T cell immunoglobulin mucin-3), a homeostasis-maintaining molecule of the immune system expressed on the surface of CD4^+^ T type 1 helper cells (Th1) and CD8^+^ T type 1 cytotoxic cells, monocytes/macrophages, dendritic cells (DC), and mast cells [28], the lectin protein CLL-1 regulating cell activation during inflammation and CD32, an immune-activating immunoglobulin Fc receptor family member showing broad expression on hematopoietic cells [29,30]. Furthermore, the interleukin-2 receptor alpha-chain CD25 commonly expressed on activated and regulatory T cells, but also found on resting memory T cells [31], and CD123, the interleukin-3 receptor (IL-3R) alpha chain, which is part of the IL-3R system that includes interleukin-5 receptor (IL-5R) and granulocyte-macrophage colony stimulating factor receptor (GM-CSFR), are also found on LSC. While interleukin 2 is important for survival, activation, and proliferation of T cells, the IL-3R system influences proliferation, survival, and differentiation of hematopoietic cells and is involved in immunity and inflammatory response by specifically binding respective ligands (IL-3, IL-5, and GM-CSF) [32].

Last but not least, the immunoglobulin-like and integrin-associated protein CD47 was identified as a novel AML LSC marker [33]. CD47 serves as a ligand of signal regulatory protein-1 (SIRP-1) and thereby functions as a “don’t eat me” signal, protecting LSC from macrophage phagocytosis [34].

## 4. LSC Surface Markers in CD34 Expressing Compared to CD34 Non-Expressing AML

The HSPC antigen CD34 is a well-established LSC surface marker in AML. However, approximately 30% of AML cases lack robust CD34 expression among leukemic blasts, perhaps because they originate from healthy CD34^−^ hematopoietic progenitors. The LSC compartment of these AML cases (in the following termed “CD34 non-expressing AML”) is less well studied but was shown to also contain CD34 negative LSC [18,35,36,37]. We have therefore decided to separately review LSC markers reported for CD34 expressing and non-expressing AML subtypes and their LSC (See Table 1). 

### 4.1. CD34 Expressing AML Contain CD34^+^ LSC

AML LSC were first experimentally defined as subpopulations of CD34^+^ AML cells [3]. In follow-up studies, LSC were then further enriched in this subpopulation by selection for the lack of CD38 expression, an antigen induced upon myeloid differentiation in healthy hematopoietic cells [4] functioning as a NAD+ glycohydrolase [38] or co-expression of the tyrosine phosphatase CD45RA [35,39,40], a CD45 isoform which plays a role in T cell signaling [41]. Upon isolation and injection into immunodeficient mice, LSC positive or negative for CD45RA and/or CD38 were able to induce leukemia, indicating that LSC can also be found in populations that phenotypically resemble more mature cells, such as common myeloid or granulomonocytic progenitors (CMP/GMP) [25,35,39,40]. CD45RA has been shown to potently enrich/isolate LSC compared to markers such as CD123, CCL-1, or the pan-myeloid antigen CD33 [39], however here only the CD34^+^/CD38^−^ compartment was investigated regarding CD45RA expression. Therefore, LSC might have been missed in CD34^−^ or CD38^+^ subpopulations in these studies.

Interestingly, CD45RA+ LSC were also documented to express CD123. In two independent studies, CD34^+^/CD38^−^/CD45RA^+^/CD123^+^ or CD34^+^/CD38^+^/CD45RA^+^/CD123^+^ cells were leukemogenic, highlighting the potential of CD123 as a marker capable of identifying and enriching LSC [35,40]. In line, recent studies showed that high percentages of CD34^+^/CD38^−^/CD123^+^ cells at diagnosis of AML could indicate poor prognosis in patients [42]. A retrospective analysis of more than 100 patients under 65 years of age with de novo AML indicated that higher percentages of CD34^+^/CD38^−^/CD123^+^ leukemia cells at diagnosis associate with enhanced probability of resistance to intensive chemotherapy and shorter disease-free survival [42]. A prospective study including 444 elderly AML patients confirmed the prognostic significance of CD34^+^/CD38^−^/CD123^+^ at diagnosis for the clinical outcome of patients receiving intensive chemotherapy, but not for those treated with hypomethylating agents only [43]. Since CD123 is likely not expressed on healthy HSC [44,45], it might represent an attractive candidate for immunological targeting of LSC.

Proteins of the so-called multi drug resistant (MDR) group, such as MDR1, BCRP, MDR3, MRP1 (also known as CD9), or LRP [46], are also heterogeneously expressed in AML with higher expression levels in CD34^+^CD38^−^CD123^+^ LSC. Expression of the tetraspanin protein CD9 nicely enables the discrimination of LSC from HSC. However, CD9 is also detectable on multipotent and lymphoid primed multipotent progenitors [47] and on cells from solid tumors (e.g., lung, breast, thyroid, and pancreas cancer (stem) cells [48,49]).

Finally, contradictory data exists on the expression of CD90, a multifunction cell surface glycoprotein and its involvement in LSC biology [50,51,52]. As such, it was e.g., documented that CD90 is absent on most AML blasts [52] despite its function in the maintenance of HSC both in vitro and in vivo [53], and cells lacking surface expression of this antigen were able to form colonies and lead to leukemia onset in patient-derived xenograft (PDX) assays [52]. 

### 4.2. CD34 Non-Expressing AML and Their LSC

The so-called “CD34 non-expressing AML” is known to completely lack or harbor only very low percentages of CD34^+^ subpopulations. Taussig and colleagues demonstrated that in such AML, LSC are comprised within CD34^−^ subpopulations. Among these, CD34^−^/CD38^+^ as well as CD34^−^/CD38^−^ subpopulations induced leukemia [37]. The existence of CD34- LSC was separately demonstrated by Sarry and colleagues [35]. Especially when present at very low numbers, CD34^+^ cells from such “CD34 non-expressing AML” may lack leukemogenic properties because they in fact represent contaminating cells that are of a non-malignant nature. This notion is supported by their capacities to regenerate normal hematopoiesis in xenotransplanted mice [37].

The existence of CD34 non-expressing AML (and CD34^−^ LSC) suggests that these cells may derive from more differentiated (CD34^−^) healthy hematopoietic cells. They might undergo leukemic transformation by acquisition of mutations in genes aberrantly conferring self-renewal and stem-like properties, such as mutations in nucleophosmin *NPM1*, which are commonly found in CD34 non-expressing AML, thereby leading to aberrant activation of Hox gene expression [54].

Further enrichment within CD34^−^ LSC subpopulations can be provided by analyses of surface expression levels of the transmembrane tyrosine kinase cKIT (CD117) and the natural killer cell receptor 2B4, also known as CD244 (See Table 1). cKIT+ cells alone showed various level of engraftment, but cKIT and CD244 double positive cells robustly engrafted in all AML cases [36]. Among healthy hematopoietic cells, cKIT and CD244 are expressed by GMPs. In line, CD34^−^ LSC from *NPM1* mutated AML were shown to harbor both a GMP and a stemness signature [36]. Accordingly, Goardon and colleagues demonstrated that LSC can derive from more committed progenitors regardless of their CD34 expression [40]. Furthermore, CD32 expression was shown to enrich LSC when applied in conjunction with CD38 in CD34 non-expressing AML [7]. Finally, although being expressed at higher levels in CD34-non-expressing AML, CLL-1 expression can distinguish LSC in both CD34-expressing and non-expressing AML [55,56]. Of note, CLL-1 is apparently not expressed on healthy HSC [24]. Further underscoring the complexity of these heterogeneous cellular systems, there are also data reporting that CD34^−^ LSC may develop in vivo into serially transplantable CD34^+^ and CD34^−^ cells [36], which in secondary recipients may give rise to both subpopulations. 

### 4.3. Review of Markers Capturing LSC in AML Samples Regardless of Their CD34 Expression

In the following section, we review the most robust markers that have been described to enrich LSC across all AML, regardless of their CD34 expression.

#### 4.3.1. Absence of NKG2D Ligands

Recently, we have shown that LSC suppress NKG2DL expression on their surface to avoid NK-mediated killing [6]. NKG2DL^−^ but not corresponding NKG2DL^+^ AML cells from the same patients were demonstrated to induce leukemia in PDX models as well as in in vivo syngeneic mouse leukemia models, despite the fact that both subpopulations contained similar leukemia-specific mutations.

When compared to NKG2DL^+^ cells, NKG2DL^−^ cells showed enriched *PARP1* expression [6]. Importantly, suppression of PARP1 could induce NKG2DL expression on previously NKG2DL^−^ LSC, making them sensitive to NK-mediated recognition and killing. Consistently, treatment with PARP1 inhibitors followed by allogeneic NK cell transplantation could eradicate primary human LSC in PDX assays in vivo. This is the first study to provide functional information on the relationship between stem cells and NK cell immune evasion in AML.

#### 4.3.2. GPR56

The GPR56 protein was first discovered in neural stem cells, where its mutant form associates with brain malformations [99]. Subsequent studies demonstrated that GPR56 is also expressed on HSC with long-term repopulation potential [94]. Interestingly, GPR56 expression was also detectable on AML cells and specifically on LSC. Pabst and colleagues furthermore observed various level of LSC activity in PDX assays for cells expressing different levels of CD34 and GPR56, with the double positive population showing the highest in vivo leukemia-initiating capacity [93]. Inhibition of GPR56 in leukemic cells decreased BM and tissue infiltration capacity, indicating a functional role in AML LSC. Mechanistically, GPR56 loss was associated with increased leukemic cell apoptosis and impaired ability of LSC to adhere in the BM niche in a RhoA-dependent manner, while colony formation interestingly remained unchanged [19,100,101]. Finally, targeting AML cells using a blocking anti-GPR56 antibody demonstrated anti-leukemic activity and prolonged survival in PDX assays [101].

As observed with other LSC markers, high GPR56 expression has been associated with poor clinical outcome in patients [93]. In fact, GPR56 is one of the genes that is part of the 17-genes stemness score [18], and was retrieved as the most strongly expressed gene in NKG2DL^−^ LSC [6]. The involvement of GPR56 in healthy HSC [19] might limit its relevance for AML treatment; however it remains a robust marker for distinguishing LSC from non-LSC.

#### 4.3.3. CD200

CD200, a glycoprotein from the immunoglobulin superfamily, represents the latest surface marker described to enrich LSC in both CD34-expressing and non-expressing AML [90]. In healthy blood cells, CD200 was reported to be expressed on HSPCS and other cells (Table 1) and to negatively regulate memory T and NK cells function in AML [90,102,103]. In AML cases with >10% CD200^+^ among CD45^dim^ cells, leukemic engraftment was only observed from CD200^+^ cells, while for samples with <10% CD200^+^ of CD45^dim^ cells, CD200^+^ cells gave rise to multilineage grafts, indicating contamination with healthy cells [90]. Moreover, CD200^+^ cells encompass both CD34^+^ and CD34^−^ cells and robustly enrich LSC in PDX assays from CD34 non-expressing *NPM1* mutated AML [90]. Finally, transcriptomic data confirmed a HSPC-like signature in CD200^+^ cells when compared to a myeloid-like signature in CD200^−^ cells. 

## 5. Phenotypic LSC Evolution and Intra-Patient Heterogeneity

**Figure 2:**
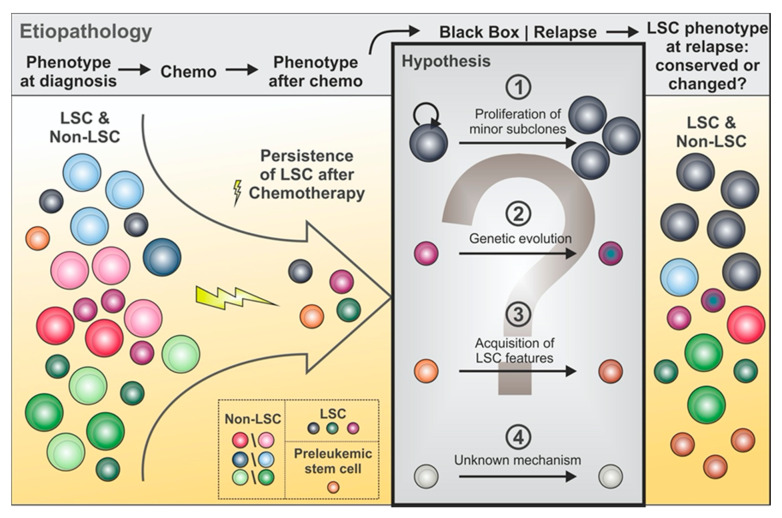
AML evolution and LSC heterogeneity after therapy. At diagnosis, various leukemic (sub)clones with corresponding LSC and non-LSC subpopulations may co-exist next to healthy and pre-malignant HSPC. Sensitivity to treatment varies between such cells, resulting in the elimination of non-LSC and potentially selection of (sub)clones and their corresponding LSC. Relapse may be driven by persistent LSC from the main (sub)clone(s) or from LSC from minor subclones that at diagnosis remained undetectable but then grow out to drive relapse. Furthermore, genetic evolution might occur conferring growth advantages to LSC and perhaps changing their surface phenotype. Finally, new leukemic clones can develop through acquisition of further mutations in the pre-malignant HSPC; disease "relapse" is in this case driven by de novo leukemic clones.

Over the last decade, many studies focused on the phenotypic and molecular characterization of LSC, with the ultimate goal of developing tools for better prediction on disease aggressiveness and improving therapy results by targeting LSC. The field has proven challenging due to the vast heterogeneity between LSC within different AML as well as within one patient during the course of the disease (Figure 2).

Markers like TIM3 [97], CD25 [60], CD32 [60], CD96 [85], and CLL-1 [104] showed LSC enriching abilities in PDX models in some, but not all AML cases. Furthermore, marker expression was noted to sometimes change during the course of the disease even within the same AML. For example, CD25^+^ LSC were shown to give rise to a progeny of CD25^−^ LSC capable of leukemic engraftment in serial transplantation assays in PDX models [61]. CD123 expression was furthermore shown to be highly variable from diagnosis to relapse in AML samples [24,25,45,55]. A possible explanation for phenotypic shifts is genetic evolution, e.g., by acquisition of novel mutations in the same leukemic (sub)clones or partially transformed HSPC (e.g., carrying pre-leukemic mutations) (Figure 2). 

This is consistent with the results documented by Becker and colleagues in comparative LSC analyses from paired samples collected from patients at diagnosis or relapse. By using CD34/CD38 or CD32/CD38 gating strategies for CD34 expressing and respectively non-expressing AML, the authors identified differences in LSC phenotype between these two time-points. For example, marker combinations that failed to identify LSC at diagnosis could indeed retrieve subpopulations with LSC activity in the corresponding relapse sample. On the molecular level, these newly engrafting subpopulations isolated from the relapse samples gained mutations in i.e., *DNMT3A*, *CDKN2A*, and differences highlighted by high-dimensional mass cytometry assays are indicative of molecular evolution [7].

Another major explanation for changes in the LSC phenotype within the same patient is (sub)clonal shifts in response to treatments. As such, initially underrepresented leukemic (sub)clones, which are hypoproliferative and show enhanced therapy resistance, may grow out under therapy, thereby becoming increasingly detectable at later time points. In contrast, less resistant (sub)clones and their LSC compartments may be preferentially eradicated by such therapies. Thus, LSC markers may be conserved or not in diagnosis versus relapse samples, reflecting these shifts in clonal dynamics [25]. Various scenarios have been reported for different AML samples and markers at diagnostic compared to relapse samples (e.g., TIM-3 [24], CLL-1 [55], GPR56: with similar [55] or higher expression [93]), in line with the possibility that relapse-driving therapy-resistant minor clones and their LSC are already present at diagnosis and then survive therapies to cause deadly relapse (Figure 2).

## 6. Association between the Genetic Background and the LSC Phenotype in AML

Several studies have linked genetic alterations with specific phenotypes. As mentioned above, CD34 non-expressing AML often show *NPM1* mutations [36,54], while this mutation has also been linked to high expression of CD123 [45]. Furthermore, high CD47 surface expression was associated with FLT3-ITD mutations [34], but not with *FLT3-TKD*, *EVI1*^high^, *NRAS*, *KRAS*, or *CEBPA* mutations, while TIM3 expression was correlated with core binding factor (CBF)-translocations, t(8;21)(q22;q22), inv(16), or *CEBPA* [104]. Recently, GMP-like LSC were linked to mutations in *CEBPA*, *DNMT3A*, and *IDH1* mutations, whereas MPP-like LSC were identified in *KRAS* and *NRAS* mutated AML. Finally, lymphoid-primed multipotent progenitor (LMPP)-like LSC were found in AML with *TP53* or *ASXL1* [105].

AML with a monosomic karyotype, CBF AML, or AML with chromosomal inversion did not show any specific phenotype, but were documented to express CD33 and CD123 at various level like other AML subgroups [63]. CBF-AML commonly associated with low CD33 expression [106], and the specific CBFB MYH11 AML showed enhanced NKG2DL expression [6]. Furthermore, strong expression of CD34 and cKIT were observed in AML with inv(16) [107]. Additionally, the presence of CD34^+^CD123^+^CD25^+^CD99^+^ subsets has been reported to be associated with FLT3 mutations in *NPM1*-positive AML [108].

### 6.1. GPR56

GPR56 was identified on LSC of high-risk AML such as *EVI1*^high^ AML [19], but also on LSC from AML with mutations in *NPM1* and *FLT3* [102], *RUNX1* or *TP53* [94]. More recently, a higher frequency of LSC phenotyped as GPR56^high^CD34^low^ cells was noted in *DNMT3A*, *NPM1*, and *FLT3-ITD* triple mutated AML, which also showed enrichment for the transcription factor hepatic leukemia factor (HLF) [109]. HLF suppression reduced the LSC content and engraftment ability by slowing cell cycle progression through *HES1*, a transcriptional repressor, and *CDKN1C*, a kinase inhibitor that negatively regulates the cell cycle.

### 6.2. CD93

CD93 is a C-type lectin connected to cellular adhesion. Its expression may regulate niche interactions and it was first described as LSC marker in chronic myeloid leukemia [110], but later shown to be also expressed on MLL-rearranged (MLLr) AML [70], specifically on the CD34^+^CD38^−^ compartment. In contrast, healthy cells and leukemic cells from other AML subtypes did not significantly express CD93. When compared to the CD93^−^ counterpart MLLr AML cells, CD93^+^ cells were shown to possess enhanced abilities to induce colonies in colony forming unit assays and leukemia in PDX models. Mechanistically, CD93 expression regulates LSC differentiation, self-renewal capacity, and in vivo progression by modulating the cell cycle inhibitor *CDKN2B* [71].

### 6.3. CD26

Recently, CD26, a multifunctional ectoenzyme expressed on T cells, was linked to AML bearing FLT3-ITD mutations [25]. The majority of FLT3-ITD positive AML cases also showed higher CD25 levels compared to patients with wild-type FLT3. Interestingly, wild-type FLT3 AML cases did not express CD26 on the surface, but in fact, harbor CD26^−^ LSC. FLT3 ITD positive AML also displayed higher expression of CD33 and CD123 compared to AML LSC with wild-type FLT3 [25], as also documented by other research groups [24,64].

Collectively, genetic backgrounds and respective phenotypes are in parts linked in AML [105] and may be therapeutically exploitable in some cases (e.g., CD93 targeting in MLLr AML [82] or CD26 in FLT3 ITD AML [25]). Specific phenotypes might be the result of mutated genes inducing certain surface markers. Alternatively, phenotypes might reflect the cells of origin with different susceptibility for selected mutations.

## 7. Therapeutic Targeting of LSC

CD33 belongs to the immunoglobulin superfamily and is a member of the sialoadhesin family of cellular interaction molecules. It is expressed on healthy HSPC and myeloid lineage cells [24], with some expression also detectable on peripheral blood lymphocytes and NK cells. 

In patients with AML, CD33 is expressed on the majority of leukemic blasts and found on both bulk AML and the LSC [63,111,112]. CD33 has been used as a target in AML, alone or in conjunction with CD123 [113,114,115]. Relapse was still observed in these patients, possibly due to escape mechanisms such as absence or downregulation of CD33 expression in LSC [106,116]. Moreover, because of the overlapping expression on healthy cells, targeted therapy against CD33 with the antibody drug conjugate gemtuzumab ozogamicin (GO) showed several adverse events such as hepatotoxicity, cardiotoxicity, hemorrhages, or infections [117,118,119] and was after first studies withdrawn from clinical use. More recently, GO obtained re-approval for the use in specific clinical applications in patients with AML [119,120].

CD47 is currently also studied as a therapeutical target in AML. Majeti and colleagues initially showed that blockade of the CD47-SIRPα axis using a monoclonal CD47 antibody can induce macrophage-mediated LSC killing and suppress in vivo leukemia development in experimental models [34]. Another in vivo study demonstrated that LSC clearance by macrophage-mediated phagocytosis is dependent on SIRPα signaling [121]. Using a SIRP-Fc fusion protein, the authors showed that disruption of the CD47-SIRPα interaction enhanced phagocytosis, leading to impaired leukemic engraftment of AML cells in NOD/SCID mice. Treatment with a humanized monoclonal antibody against CD47 furthermore eradicated AML LSC, leading to long-term disease-free survival in PDX assays [122]. This antibody has now entered clinical trials in patients with AML and solid tumors. The significance of CD47 as a target in AML therapy was validated in further reports [123,124]. Recent clinical data from another phase 1B study indicates that a combination of vincristine and magrolimab, a first-in-class antibody targeting CD47, may be effective in the treatment of AML and MDS [125]. Lately, enhanced CD47 expression was linked to CD123 expression and shown to be responsible for drug resistance in AML that could be overcome by treatment with the histone deacetylase inhibitor Romidepsin [126]. However, phase 1 trials using monoclonal anti-CD47 antibodies were terminated due to insufficient activity (CC-90002, NCT02641002) [127], life threatening side effects (Ti-061, 2016-004372-22; Hu5F9-g4, NCT02678338), or anemia (due to CD47 expression on red blood cells [128]). Results from other currently recruiting clinical trials are underway.

The transmembrane glycoprotein CD44, known to bind hyaluronan, selectins, and osteopontin, displays a plethora of functions in healthy and diseased tissues [69] and has been targeted therapeutically in AML before it was described as an LSC marker. Overall, CD44 shows higher expression in AML cells compared to healthy HSC and displays several splice variants that are heterogeneously distributed among AML cases. High expression of CD44-6v especially correlates with shorter survival in patients with AML [71,129]. Treatment with CD44 antibodies was shown to inhibit proliferation and induce differentiation and apoptosis in AML cells [130,131,132,133]. Later on, CD44 targeting was reported to also eradicate AML LSC in PDX assays by impacting LSC trafficking to BM niche [70]. Future research will show whether the therapeutic effect of anti-CD44 antibodies may be potentiated by combinatorial application with other drugs [134].

Targeting CD123 has also been reported to show anti-leukemic effects in preclinical as well as clinical studies. Jin et al., for example, demonstrated that the use of a neutralizing CD123 antibody was able to inhibit leukemogenicity in PDX assays [135]. Ex vivo treatment of bulk AML or LSC with a neutralizing-antibody or direct injection at different time points of this antibody in mice reduced engraftment and improved survival in different animal models. This decrease is linked to a reduced homing combined with an antibody-dependent cell-mediated cytotoxicity (ADCC) effect. On the molecular level, CD123 blockade reduces proliferation and survival of in vitro cultured AML cells [135]. CD123 also helps clinicians to monitor disease outcome, in which CD34+CD38-CD123+ LSC levels are higher in the non-complete remission group [136] and represents an interesting target in cancer treatment (reviewed elsewhere [137]). Clinical trials targeting CD123 were initiated, but unfortunately in several cases, suspended ahead of schedule (i.e., NCT02715011, NCT02113982, or Talacotuzumab, e.g., due to serious adverse events). Interestingly, the single-agent flotetuzumab, an investigational CD123 × CD3 bispecific DART protein, has shown evidence of clinical activity in a Phase 1 study of relapsed/refractory (R/R) AML [138,139]. Further clinical trials using CD123 CAR T cells were initiated (NCT02159495, NCT04230265) with so far promising results [140,141].

TIM-3 and CLL-1 are additional surface proteins which make interesting targets, because they are both absent on healthy HSCs (Table 1). Clinical trials with promising results are underway or were performed with agents targeting these molecules (TIM-3, phase 1b clinical trial, NCT03066648; CLL-1, [142]).

Finally, the tumor necrosis factor receptor and LSC marker CD70 may also serve as a potential target molecule in AML. Transiently upregulated on immune cells upon activation, CD70 is otherwise not expressed in normal tissues [143]. In AML, CD70 expression was reported to promote blast stemness [77]. Treatment with cusatuzumab, a human αCD70 monoclonal antibody with enhanced antibody-dependent cellular cytotoxicity activity, was recently shown to hold anti-leukemic activity in in vitro and in vivo PDX assays. In a phase 1 study, cusatuzumab alone or in combination with azacitidine showed pronounced efficacy in previously untreated AML patients or patients that are unfit for intensive chemotherapy [144]. Further clinical phase 2 and 3 trials using these approaches are underway.

## 8. Concluding Remarks

LSC and their biology gained great interest in the last decades, since it is now well accepted that efficient targeting of this subpopulation is essential to achieve cure in patients with AML. Defining the surface markers that reliably identify LSC is a critical goal, since it enables further investigations of these subpopulations, monitoring of the clinical course, and the development of novel immunotherapy strategies targeting surface antigens in LSC.

Next to their close molecular relationship to HSPC (Figure 1), the greatest challenge in targeting LSC is their profound heterogeneity among patients as well as within the same patient (see Table 1 ‘Percentage of AML Patients Expressing the Marker` and Figure 2). The establishment of marker combinations may be required for both diagnostic [22,24,26,145,146] and therapeutic purposes (i.e., targeting CD123/CD47 [126], CD33/TIM3 [24], CLL1/TIM3 [24], CCL1/CD56 [104], or CD33/CD123 [114]). Selected genetic lesions may induce the expression of specific surface antigens (e.g., CD93 on MLL-r AML LSC [82]), which may hold great promise, however, currently remains exceptional and only applicable to rare AML subtypes.

Novel technical developments allowing high-throughput screening of low amounts of cells on both transcriptome [18,25,147], proteome [147,148], and surface antigen level [25] may provide further valuable insights into LSC surface antigens (e.g., identification of the fatty acid translocase CD36 and the type 2 C-lectin receptor CD69 via single-cell RNA sequencing [67]).

Finally, although some surface markers sound appealing as AML targets (e.g., CD123 or CD47), it still remains challenging to safely target them in patients, as observed by many trials still in a lagging phase or stopped due to severe toxicities. Personalized approaches involving multi-antigen detection and validations during disease evolution, or alternative strategies that e.g., induce antigen expression to make LSC targetable for immunological therapies [6] may hold promise for efficient LSC targeting in the future.

## Figures and Tables

**Table 1:** Non-comprehensive list of human markers that can be found on LSC and their (potential) expression on the cell surface of other healthy blood cells. Highlighted in grey are the markers expressed on the cell surface of LSC from both CD34-expressing and non-expressing acute myeloid leukemia (AML). In white: markers only demonstrated to play roles in LSC from CD34 expressing AML. N.D: Not described/MPP: multipotential progenitor/MEP: megakaryocyte–erythroid progenitor).

Antigen	Percentage of AML Patients Expressing the Marker	Expression on Non-LSC	Expression on HSC	Expression on Other Healthy Blood Cells	Function in Healthy Conditions	References
CLL-1	92	Yes	No	Monocytes, granulocytes, CMP, GMP	Modulates the activation state of cells during inflammation processes	Bakker et al. 2004 [57] Jiang et al. 2018 [58] Daga et al. 2019 [55] Marshall et al. 2006 [29]
CD9	40	Yes	No	Monocytes, macrophages, granulocytes, DC, endothelial cells, B, T, and NK cells	Cell migration, adhesion, activation,	Brosseau et al. 2018 [59] Touzet et al. 2019 [47] Paprocka et al. 2017 [46]
CD25	10–25	Yes	No	T cells and regulatory T cells	Important role for T cells survival	Saito et al. 2010 [60] Kageyama et al. 2018 [61] Triplett et al. 2012 [31]
CD26	N.D	Yes	No	T, B, NK, and myeloid cells	T cell activation and proliferation, cell adhesion, metabolism	Herrmann et al. 2020 [25] Klemann et al. 2016 [62]
CD32	35	Yes	No	Monocytes, B and T cells	Immune cell activation	Saito et al. 2010 [60] Anania et al. 2019 [30]
CD33	88	Yes	Yes	Myeloid cells, lymphocytes, NK cells, MPP, GMP, MEP	Modulates inflammatory and immune responses by reducing tyrosine kinase dependent pathways	Ehninger et al. 2014 [63] Liu et al. 2007 [64] Laszlo et al. 2014 [65] Haubner et al. 2017 [24]
CD34	70	Yes	Yes	Mast cells, eosinophils, neurons, fibrocytes	Regulates cell differentiation, adhesion, trafficking and proliferation	Quek et al. 2016 [36] Engelhardt et al. 2002 [16] Nielsen et al. 2008 [17]
CD36	N.D	Yes	No	Platelets, monocytes, adipocytes	Fatty acid uptake, angiogenesis, PRR recognition	Silverstein et al. 2009 [66] Sachs et al. 2020 [67] Herrmann et al. 2020 [25]
CD38	5–55 (FAB subtypes)	Yes	No	T and B cells, monocytes, NK, granulocytes, platelets, red blood cells	Regulates calcium levels and NAD+ homeostasis	Hogan et al. 2019 [38] Sarry et al. 2011 [35] Goardon et al. 2011 [40] Keyhani et al. 2000 [68]
CD44	N.D	Yes	Yes	T cells, mesenchymal cells, ectodermal cells, neuron-like cells	Cell adhesion molecule, cellular signaling	Ponta et al. 2003 [69] Jin et al. 2006 [70] Bendall et al. 2000 [71] Herrmann et al. 2020 [25]
CD45RA	N.D	Yes	Yes	T and B cells	CD45 isoform, cell signaling	Kersten et al. 2016 [39] Goardon et al. 2011 [40] Sarry et al. 2011 [35] Holmes 2006 [41]
CD47	N.D	Yes	Yes	Various healthy cells	“don’t eat me” signal on cells in order to prevent inappropriate phagocytosis	Majeti et al. 2009 [34] Jaiswal et al. 2009 [33] Sick et al. 2012 [72]
CD56	Up to 20	Yes	No	DC, T and NK cells	Linked to NK cytotoxicity	Van Acker et al. 2017 [73] Sasca et al. 2019 [74] Herrmann et al. 2020 [25]
CD69	N.D	N.D	No	T cells	T cell differentiation, tissue retention, and metabolic reprogramming	Cibrián et al. 2017 [75] Sachs et al. 2020 [67] Herrmann et al. 2020 [25]
CD70	N.D	Yes	No	DC	T and B cell activation	Riether et al. 2015 [76] Riether et al. 2017 [77] Borst et al. 2005 [78]
CD90	40 (in elderly patients)	Yes	Yes	Fibroblasts, neurons, endothelial cells	Maintenance of HSC, cell adhesion, matrix adhesion	Buccisano et al. 2004 [79] Blair et al. 1997 [52] Brendel et al. 1999 [50] Kisselbach et al. 2009 [80] Craig et al. 1993 [53]
CD93	N.D	N.D	No (only on CD34-HSC)	Myeloid and endothelial cells	Mechanism in innate host defense	Bohlson et al. 2008 [81] Iwasaki et al. 2015 [82] Sumide et al. 2018 [83]
CD96	27	Yes	Only 5%	T and NK cells	Inhibits NK and T cells	Fatlawi et al. 2016 [84] Georgiev et al. 2018 [27] Hosen et al. 2007 [85]
CD117	87	Yes	Yes	GMP	Promotes HSC growth by binding the stem cell factor	Sperling et al. 1997 [86] Geissler et al. 1991 [87] Quek et al. 2016 [36] Wells et al. 1996 [88]
CD123	97	Yes	No	Basophils, plasmacytoid DC	Proliferation, survival, activation, and differentiation by binding respective ligand	Yu et al. 2016 [88] Guthridge et al. 1998 [32] Bras et al. 2019 [45] Haubner et al. 2019 [24] Al-Mawali et al. 2017 [44]
CD200	N.D	Yes	Yes	Myeloid, T and B cells	Immunoregulatory molecule	Ngwa et al. 2019 [89] Ho et al. 2020 [90]
CD244	N.D	Yes	Yes	GMP, HSPC, granulocytes, monocytes, DC, NK and T cells	Regulates NK, T, and DC activation state	Zhang et al. 2017 [91] Haubner et al. 2019 [24] Quek et al. 2016 [36] Agresta et al. 2018 [92]
GPR56	N.D	No	Yes	Central nervous system, T cells	Frontal cortex development, NK inhibition, cell migration, HSC generation	Pabst et al. 2016 [93] Daga et al. 2019 [55] Kartalaei et al. 2015 [94] Huang et al. 2018 [95]
NKG2DL (its absence defines LSC)	Highly variable	Yes	No	Not expressed on healthy cells	Upregulation of NG2DL on malignant or virus-infected cells resulting in their clearance by NK cells	Paczulla et al. 2019 [6] Zingoni et al. 2018 [96]
TIM-3	98	Yes	No	T cells, monocytes, macrophages, DC, and mast cells	Homeostasis-maintaining molecule of the immune system	Jan et al. 2011 [97] Haubner et al. 2019 [24] Kikushige et al. 2010 [98] Han et al. 2013 [28]
